# Impacts of student debt on the professional and personal lives of genetic counselors: A 10‐year perspective

**DOI:** 10.1002/jgc4.70013

**Published:** 2025-04-30

**Authors:** Holly L. Greer, Elizabeth M. Petty, Michael R. Lasarev, Ashley Kuhl

**Affiliations:** ^1^ Department of Pediatrics, School of Medicine and Public Health University of Wisconsin–Madison Madison Wisconsin USA; ^2^ Department of Biostatistics & Medical Informatics, School of Medicine and Public Health University of Wisconsin‐Madison Madison Wisconsin USA; ^3^ Present address: Cleveland Clinic Cleveland OH USA

**Keywords:** career choices, genetic counseling, life choices, personal impacts, professional impacts, student loan debt

## Abstract

The financial landscape for those entering the genetic counseling profession has shifted significantly regarding salaries and educational costs since the 2014 study by Kuhl et al. Limited information is available regarding whether and how educational costs and student debt impacts have changed over time for genetic counselors. An updated web‐based survey of 317 recent genetic counseling program graduates was conducted to address this gap. Overall, educational costs, total debt, and salaries showed increases from the prior study, and many factors measuring the impact of genetic counseling graduate student debt showed little to no change. Ninety‐six percent (*n* = 222/232) of respondents still reported feeling burdened by their student debt, and 45% (*n* = 105/232) reported experiencing more hardship than anticipated, though most respondents were once again satisfied with their genetic counseling education. Student debt influenced many lifestyle choices to a strikingly similar degree, and a large majority still said that this debt did not change their career path post‐graduation. Several factors did not significantly impact reported hardship, including having received financial help from family members or financial counseling before starting a genetic counseling program. Total debt and starting salaries had medians of $50,000 to $75,000 and $70,000 to $75,000, respectively, again exceeding expert recommendations that student debt payments do not surpass 10% of the borrower's projected gross monthly income. These results suggest ongoing burden and hardship caused by student debt and similar impacts on professional and personal life choices compared to a decade ago. In response to these ongoing issues, we suggest ways to alleviate this burden and increase financial transparency and access to the profession for future cohorts.

**What is known about this topic**

The 2014 study by Kuhl et al. revealed that genetic counseling program graduates felt burdened by high student debt loads that, on average, outpaced debt‐to‐income recommendations from financial experts. Despite this debt burden and its impacts on personal and professional life choices, graduates at that time reported overall satisfaction with their genetic counseling education.

**What this paper adds to this topic**

This paper aimed to re‐evaluate the financial landscape and overall perceived burden and hardship caused by student debt for recent genetic counseling program graduates. This updated information will help highlight ways to increase financial transparency and suggest efforts toward improved access and diversity within the genetic counseling profession.

## 1 INTRODUCTION

Education‐related costs of professional healthcare programs influence career and lifestyle choices among graduates as described in discipline‐specific studies, though genetic counseling‐specific information has been limited since a 2014 study by Kuhl et al. For physician assistant (PA) graduates, financial factors have significantly influenced job choices. Specifically, PA students who felt educational debt strongly influenced their career choice were more likely to choose a job in primary care over specialty, potentially due to additional loan forgiveness and repayment options. Inversely, students who described income potential as moderately or strongly influencing their choice of specialty had higher odds of choosing a specialty position over primary care (Twombly et al., 2019). Similarly, a 2022 survey of Doctor of Public Health graduates found that student debt impacted career choices, potentially preventing recent graduates from working in the public or non‐profit sectors where wages are lower (Park & Coles, 2022). Doctor of Physical Therapy (DPT) students have also reported that student debt impacted their program and job choices, as well as decisions to forgo or delay important life milestones. A June 2020 survey of DPT graduates reported that 70% experienced mid‐ to high‐level anxiety regularly due to their student debt, often using words, such as “burden” and “trapped,” to describe feelings regarding their student loans (American Physical Therapy Association, 2022).

The financial burden associated with post‐secondary education also poses possible barriers to increased access and diversity within Medicine and allied health professions. Certain segments of the DPT population are more burdened by student debt, with rising education costs disproportionately affecting students from minority, low socioeconomic status (SES), and first‐generation families (American Physical Therapy Association, 2022). A 2023 study by Faiz et al. found that underrepresented MCAT examinees faced increased educational and financial barriers, including outstanding loans. Students with low SES entering the medical field have also reported feeling the burden of medical school costs disproportionately more than their high SES counterparts (McMichael et al., 2022). Many allied health and medical programs have implemented initiatives to address the cost of training. The Association of American Medical Colleges has an assistance program to aid students with application fees at a set income level, and Weill Cornell Medicine saw the percentage of applicants from underrepresented and low SES groups increase after launching a program to eliminate debt for medical students with demonstrated financial need in 2019 (Kang & Ibrahim, 2020). Within the genetic counseling field, some efforts to reduce financial barriers have included program scholarships and registration fee waivers funded by the Genetic Counseling Educators Association (GCEA), formerly known as the Association of Genetic Counseling Program Directors, for the GC Admissions Match (National Matching Services Inc, 2024). The Alliance to Increase Diversity in Genetic Counseling (AID‐GC) is also working to provide scholarships, fellowships, mentorship, and curriculum focused on diversity and inclusion, though this resource is currently limited to five participating GC programs, thus limiting influence (Alliance to Increase Diversity in Genetic Counseling, n.d.).

For those who can pursue these professions despite the high costs and financial barriers, many require aid. Federal financial aid available to graduate students included up to $20,500 each year in Direct Unsubsidized Loans in 2023, with Direct PLUS Loans available to students to cover the remainder of the cost of attendance for their program. The Federal Perkins Loan Program, which offered low‐interest federal student loans for undergraduate and graduate students with exceptional financial need, was discontinued in September 2017 (US Department of Education, n.d.‐b).

Many professional healthcare program graduates may pursue Public Service Loan Forgiveness (PSLF), a government program formed to provide student debt relief to professionals with qualifying loans who work full time for eligible employers (US Department of Education, n.d.‐a). Additional efforts to address student debt burden from the federal government have included expanded forgiveness opportunities and reformed income‐driven repayment plans (Council of Economic Advisers, 2024).

In addition to government‐level reform, the onset of the COVID‐19 public health emergency in March 2020 significantly impacted student loan borrowers in the United States. These pandemic‐related financial impacts were similar to those experienced by graduates (including the 2013 survey cohort) during and after the Great Recession of 2008 (Larrimore et al., 2022). The federal government in March 2020 implemented several measures to provide relief to federal student loan borrowers, including temporary payment forbearance and 0% interest rates on outstanding balances, which ended in September 2023 (Case et al., 2022; Federal Student Aid, n.d.).

### 1.1 Purpose of study

Since the initial 2014 study by Kuhl et al. outlining impacts of student debt on genetic counselor (GC) professional and personal choices, the financial landscape for those newly entering the profession has shifted significantly. GC salaries rose about 50% from 2013 to 2023 (National Society of Genetic Counselors, 2013; National Society of Genetic Counselors, 2023). This salary increase was in the setting of a 31% inflation increase based on the annual average consumer price index for all urban consumers (CPI‐U) in 2013 and 2023 (Federal Reserve Bank of Minneapolis, 2024). Additionally, US News & World Report published data in September 2023 that outlined growth in tuition costs at research‐oriented national universities that offer bachelor's, master's, and doctoral degrees, which showed approximate increases of 30% and 40% at public and private institutions, respectively, from 2013 to 2023 before inflation adjustment (Kerr & Wood, 2023). Despite these economic shifts, updated information regarding how these factors affect GCs in today's financial landscape has been limited, and it is unclear whether overall borrowing practices continue to exceed expert recommendations as they did in 2013.

The average starting salary for new graduates rose to $81,373 in 2023 (National Society of Genetic Counselors, 2024). With an average starting salary of $53,900 reported by participants in 2013, this represents a 51% increase over 10 years. Due to the evolving economic landscape in the United States, a current exploration of the financial impact of student debt on the professional and personal decisions of GCs is needed. With rising costs of living and education, as well as persistent effects of student debt among other healthcare professions, little may have changed since 2013 regarding debt‐to‐income ratios and the financial burden reported by GC program graduates. This study aimed to understand impacts of student debt has on the GC profession and suggest ways to address financial barriers as the profession seeks to diversify and increase access to GC services.

## 2 METHODS

### 2.1 Participants

This is an observational cross‐sectional study targeting recent graduates of North American GC programs. Participants were recruited via email listservs through the National Society of Genetic Counselors (NSGC) and the GCEA. A listing request was submitted to NSGC for inclusion in their biweekly Student Research Survey Program email, including the survey link and eligibility criteria. Program directors were asked via the GCEA listserv to forward the survey to alumni of their program who graduated within the last five years (2019–2023). A reminder email was sent to each organization two weeks after the initial request. The five‐year timeframe was selected as these graduates would have experienced similar economic climates and to ensure more accurate recall. Links to the survey were also posted on social media (LinkedIn and Reddit). Eligibility criteria were listed on the first page of the survey.

Participants were asked to complete the survey via Qualtrics, an online web‐based survey (www.qualtrics.com) to assess updated data and views regarding their student debt. All data were collected anonymously and stored electronically through Qualtrics. The survey was open for responses for four consecutive weeks (October to November 2023), with social media posts spanning the same period.

### 2.2 Instrumentation

Survey questions were adapted from the previous study completed by Kuhl et al. (2014), and data from the prior survey was used for comparison across years (2013 and 2023).

Like the prior study, this survey contained three main sections to assess the following: (1) amount of educational debt accumulated before starting a GC program and impact on program choice; (2) amount of debt accumulated during GC education and impact on career and later life choices; and (3) demographic information. The 82 questions comprised a combination of 5‐point Likert rating scale (17 items), dichotomous (19 items), categorical (39 items), and open‐ended (7 items) response options. Respondents were not required to answer every question, and “not applicable” was included as an option within matrix questions for variables that may not apply to every participant. Skip logic was used to prevent participants from answering unnecessary questions.

The survey was piloted via Qualtrics by six GCs and graduate students at the University of Wisconsin–Madison to assess questions for clarity, duration, and technical difficulties. The survey was then reviewed and approved (classified as exempt) by the Education Research and Social & Behavioral Science IRB at the University of Wisconsin–Madison.

### 2.3 Data analysis

Data were tabulated and summarized using frequencies and percentages. Chi‐squared or Fisher's exact tests were used to explore associations involving unordered categorical factors with differences reported as percentage points of separation. Ordinal outcomes were compared between groups using Wilcoxon rank‐sum tests; if the ordinal outcome was compared among three or more groups, then the Kruskal–Wallis test was used with subsequent pairwise comparisons performed using the Steel‐Dwass non‐parametric multiple comparison procedure. Spearman's rank correlation coefficient was used to test for associations involving pairs of ordinal outcomes (i.e., ordinal‐by‐ordinal associations). Confidence intervals (95% coverage) for Spearman's rank correlation coefficient were derived using jackknifed empirical‐likelihood procedures described by Wang and Peng (2011). Wilcoxon's measure of ordinal preference was used to describe the difference between groups with respect to a given ordinal outcome and represents the probability that a randomly chosen observation from one group would be higher than a randomly chosen observation from the other, with a probability of 50% signifying no difference (no ordinal preference). Comparison of effect measures (Spearman's rho or Wilcoxon's measure of preference) from 2023 to 2013 were completed using the method of variance estimate recovery (MOVER) to construct a 95% confidence interval for the difference between the two measures (Zou & Donner, 2008). If the confidence interval for the difference of effect measures from the two separate years included 0, then there was no statistically significant difference between years for the effect measure. If the confidence interval for the difference failed to cover 0, then effect measures significantly differed by year at the 0.05 level. All confidence intervals for the difference between effect measures were computed as the effect for 2023 minus the effect for 2013 (later minus earlier). Analyses were performed using Stata (version 17; StataCorp LLC, College Station, TX) or R (version 4.4.0; https://cran.r‐project.org/). No inflation adjustment was used due to the varying timeframes (and varying CPI‐U rates) in which respondents among each cohort would have taken out loans, paid tuition, and established salaries. Changes in these metrics over time were instead reported in the setting of the overall change in annual CPI‐U rates between 2013 and 2023.

## 3 RESULTS

### 3.1 Response rate and demographics

A total of 317 online surveys were completed, representing a minimal response rate of 30% (*n* = 317/~1068) based on the number of 2019–2023 graduates reported by the NSGC 2023 Professional Status Survey (PSS). Since participants were not required to answer every question, the response rate was lower for some variables. Participant demographics are outlined in Table 1. Most participants identified as female (87%), White (81%), and between ages 25 and 29 (68%). Most had no children or dependents (90%).

**TABLE 1:** Participant demographic Information (2023).

Demographic	*N*	%
Year of graduation (*N* = 317)		
2019	47	15
2020	51	16
2021	58	18
2022	75	24
2023	86	27
Age (*N* = 274)		
20–24	20	7
25–29	187	68
30–34	55	20
35–39	7	3
40–44	4	1
Over 45	0	0
Prefer not to answer	1	0
Gender identity (*N* = 274)		
Female	239	87
Male	23	8
Non‐binary	7	3
Other	2	1
Prefer not to answer	3	1
Transgender^a^	2	1
Racial/ethnic group (*N* = 274)		
White (not Hispanic or Latine)	222	81
Asian or Pacific Islander	15	5
Hispanic/Latine/Spanish	14	5
Biracial/multi‐racial	14	5
African American/Black	4	1
Other	3	1
Prefer not to answer	2	1
Genetic counseling field (*N* = 265)		
Clinical	232	88
Research	29	11
Diagnostic laboratory	25	9
Education and policy	17	6
Commercial	8	3
Public health	2	1
Other	1	0
Relationship status (*N* = 274)		
Married	86	31
Never been married	96	35
Live with someone as couple/partner	90	33
Divorced	1	0
Separated	0	0
Widowed	0	0
Prefer not to answer	1	0
Residence (*N* = 274)		
Renting or Leasing	187	68
Own a home or paying a mortgage	57	21
Live with parents/guardians	13	5
Other	13	5
Live with other family	3	1
Prefer not to answer	1	0
Children/dependents (*N* = 273)		
0	245	90
1	15	5
2	11	4
3	1	0
4	1	0
5 or more	0	0

^a^ Included in total count for those who identify as male/female.

The fraction of respondents who identified as female decreased by 5 (95% CI: 1.6–9.7; *p* = 0.005) percentage points from 2013 (96% in 2013 vs. 91% in 2023). The distribution of racial/ethnic groups also differed between years (*p* < 0.001). In particular, the percentage who self‐identified as White (not Hispanic/Latine) decreased 11 percentage points (92% in 2013 vs. 81% in 2023; 95% CI: 6.2–17.0; *p* < 0.001). A four‐percentage point increase was seen in those who identified as Asian/Pacific Islander (*p* = 0.008), those with Hispanic/Latine/Spanish identity (*p* = 0.007), and respondents with Bi‐/Multi‐racial identities (*p* = 0.028).

### 3.2 Undergraduate & pre‐genetic counseling program experience

Forty percent of participants who could recall their amount of debt reported no undergraduate student debt (*n* = 124/307), similar to the 47% (*n* = 189/405) who reported the same in 2013 (*p* = 0.095). Among those with at least some debt, the median amount in 2023 was $30,000 to $35,000, with the overall median (including those who reported $0 of undergraduate student debt) being $10,000 to $15,000. Participants with undergraduate debt reported a median of $30,000 to $35,000. Before adjusting for inflation, total undergraduate debt was higher in 2023 compared with 2013 (*p* < 0.001).

Thirty‐three percent of respondents in 2023 (*n* = 92/282) said previous student debt was somewhat or extremely important in influencing when they attended their GC program; a 12‐percentage point increase from 2013 (21%; *n* = 79/381; *p* < 0.001). Over half (56%; *n* = 157/282) of respondents from 2023 shared that previous student debt was somewhat or extremely important in influencing which GC program they attended, a 14‐percentage point increase from 2013 (41%; *n* = 158/381; *p* < 0.001). Forty‐nine percent of those from 2023 said prior student debt did not influence their decision to pursue GC graduate training (*n* = 137/282), though 26% said it was somewhat or extremely important in making this decision.

When considering factors used to determine which GC program participants ranked highly or attended, 86% (*n* = 248/287) indicated that program cost was at least somewhat influential in their choices, as well as cost of living/housing (80%), and financial assistance available (68%; Table 2). For the 2023 cohort, those who said program cost and living/housing were highly influential on program choice generally spent less on tuition and fees (*r*_s_ = −0.37, *p* < 0.001 and *r*_s_ = −0.21, *p* < 0.001, respectively), indicating that students who considered/ranked programs heavily based on financial factors generally attended more inexpensive programs. Participants were also asked to identify the single most influential factor in determining which GC program they ranked highly or attended. Location was deemed most influential by the largest percentage of respondents (30%; *n* = 85/287), followed by program cost (23%), and program assistance available (11%; Table 2). Location was also rated as most influential in determining program choice 10 years ago (38%; *n* = 157/410), followed by training program cost (15%), and training program structure/curriculum (15%). The percentage who claimed location was most important decreased 9 (95% CI: 1.5–15.6, *p* = 0.018) percentage points from 2013 (38%) to 2023 (30%), while the percentage who claimed cost as the most important factor increased 8 percentage points (15% vs. 23%; 95% CI: 1.9–13.9, *p* = 0.009).

**TABLE 2:** Pre‐genetic counseling program training experience (2023).

Question/response	*n*	%
Approximate debt accrued during undergraduate education (*N* = 309)		
No debt	124	40
Less than $5000	6	2
$5000 to $9999	15	5
$10,000 to $14,999	13	4
$15,000 to $19,999	11	4
$20,000 to $24,999	20	6
$25,000 to $29,999	19	6
$30,000 to $34,999	14	5
$35,000 to $39,999	9	3
$40,000 to $44,999	7	2
$45,000 to $49,999	6	2
$50,000 to $54,999	7	2
$55,000 to $59,999	4	1
$60,000 to $64,999	4	1
$65,000 to $69,999	1	0
$70,000 to $74,999	2	1
$75,000 to $79,999	5	2
$80,000 to $84,999	6	2
$85,000 to $89,999	3	1
$90,000 to $94,999	8	3
$95,000 to $99,999	1	0
$100,000 or more	22	7
Do not remember	2	1
Importance of previous student debt in influencing when you attended a genetic counseling program (*N* = 282)		
Not at all important	113	40
Somewhat unimportant	37	13
Neither important nor unimportant	40	14
Somewhat important	61	22
Extremely important	31	11
Importance of previous student debt in influencing which genetic counseling program you attended (*N* = 282)		
Not at all important	68	24
Somewhat unimportant	27	10
Neither important nor unimportant	30	11
Somewhat important	84	30
Extremely important	73	26
Most influential factor influencing program ranked highly or attended (*N* = 287)		
Location	85	30
Cost of program	65	23
Program structure/curriculum	32	11
Program leadership	22	8
Exposures to certain clinical specialties	16	6
Reputation of Institution or Program	14	5
Other	12	4
Financial assistance available	8	3
Exposure to certain patient population(s)	8	3
Interactions with current students	8	3
Cost of living/housing	7	2
Faculty interaction with students	7	2
Global/international opportunities available	1	0
Length of program	1	0
Research opportunities available	1	0
How influential given factors were in determining whether you ranked highly or attended a program (*N* = 287)		
Cost of program		
Not at all influential	7	2
Somewhat uninfluential	16	6
Neither influential nor uninfluential	16	6
Somewhat influential	117	41
Extremely influential	131	46
Cost of living/housing		
Not at all influential	17	6
Somewhat uninfluential	19	7
Neither influential nor uninfluential	21	7
Somewhat influential	135	47
Extremely influential	95	33
Financial assistance available		
Not at all influential	28	10
Somewhat uninfluential	22	8
Neither influential nor uninfluential	42	15
Somewhat influential	104	36
Extremely influential	91	32

### 3.3 Financing a genetic counseling training program

**FIGURE 1:**
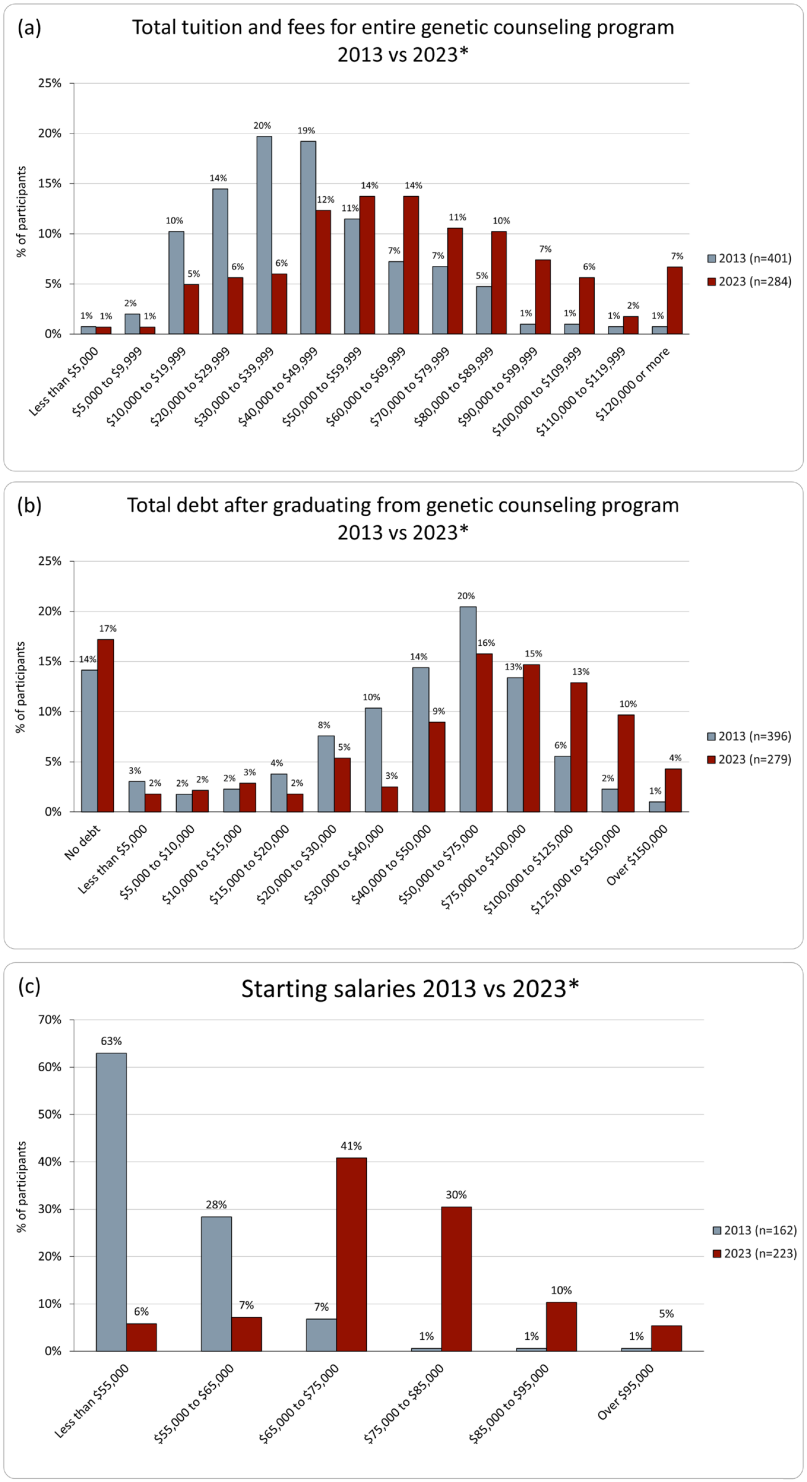
Comparisons of reported total tuition and fees (a), total debt (b), and starting salaries (c) by respondents in 2013 (gray) and 2023 (red). *Monetary values are not adjusted for inflation.

Median cost of tuition and fees for participants was $60,000 to $69,999 in 2023, compared with $40,000 to $49,999 in 2013, representing an overall increase from the prior survey before inflation adjustment (*p* < 0.001), or an overall estimated 44% increase (calculated from median range midpoints) across 10 years in the setting of a 31% increase from inflation (Figure 1a).

Participants used a variety of funding sources to finance their education, including loans and other forms of financial assistance (Table 3). Sixty‐six percent of respondents (*n* = 190/287) shared that availability of student loans was extremely important in allowing them to attend their training program. The importance of loan availability was directly associated with total program tuition and fees (*r*_s_ = 0.25, *p* < 0.001). For those who utilized federal student aid, Federal Direct Unsubsidized loans (79%; *n* = 174/221) and/or Federal Graduate PLUS loans (58%) were most common, which came with respective interest rates of 7.05% and 8.05% as of July 1, 2023. Primary repayment options for most respondents were either the Standard Repayment Plan (10‐year fixed monthly payments; 32%; *n* = 71/220) or the Saving on a Valuable Education (SAVE) Plan—formerly the REPAYE Plan (33%). Other federal repayment plans used by respondents are outlined in Table 3. Only 13% (*n* = 38/282) of graduates said they took out private loans. Nineteen percent of respondents (*n* = 55/284) said they did not utilize any federal or private loans to finance their genetic counseling education.

**TABLE 3:** Financing a genetic counseling education (2023).

Question/response	*n*	%
Federal loans used to finance genetic counseling education (*N* = 221)		
Federal Direct Unsubsidized loans	174	79
Federal Graduate PLUS loans	128	58
Other	20	9
Federal Perkins loans	7	3
Do not remember	7	3
Private loans used to finance genetic counseling education (*N* = 38)		
I took out private education loans	32	84
No private loans were used for my education	4	11
Parent(s)/family/spouse/significant other took out private education loans	3	8
Importance of loan availability to attend genetic counseling program (*N* = 287)		
Not at all important	32	11
Somewhat unimportant	7	2
Neither important nor unimportant	8	3
Somewhat important	50	17
Extremely important	190	66
Work/employment used to finance genetic counseling program (*N* = 279)		
Paid work off‐campus	89	32
I did not work during my program	82	29
Assistantships (graduate, research, teaching, etc.)	75	27
Paid work on‐campus	41	15
Federal Work‐Study (FWS) program	29	10
Paid internship	7	3
Hours worked per week during genetic counseling program (*N* = 198)		
1–5	35	18
6–10	73	37
11–15	41	21
16–20	31	16
21–25	11	6
26–30	3	2
More than 30	4	2
Effect of work on academic performance (*N* = 192)		
Greatly hurt my performance	3	2
Somewhat hurt my performance	51	27
Neither helped nor hurt my performance	99	52
Somewhat improved my performance	35	18
Greatly improved my performance	4	2
Importance of work to cover educational expenses (*N* = 189)		
Not at all important	17	9
Somewhat unimportant	23	12
Neither important nor unimportant	25	13
Somewhat important	73	39
Extremely important	51	27
Whether credit cards were used for necessary educational expenses (*N* = 280)		
Yes	61	22
No	41	15
Yes, but I never carried a balance (no interest accrued)	178	64
Grants/scholarships used to finance genetic counseling (*N* = 127)		
Program scholarship/stipend	92	72
Merit‐based university/college grants or scholarship	18	14
Private/public agency or foundation scholarship	13	10
Other	13	10
Diversity grant or scholarship	7	6
Merit‐based state grants/scholarship	5	4
Needs‐based university/college grants or scholarship	4	3
Needs‐based state grants/scholarship	1	1
Financial assistance available through program (*N* = 202)		
National meeting/conference attendance fees	132	65
Partial scholarship	106	52
Work‐study opportunities	95	47
Research funds	68	34
Travel reimbursement or assistance	59	29
TA/RA opportunities	55	27
Other	38	19
Books	30	15
Assistance with grant applications	7	3
Subsidized housing	4	2
Full scholarship	1	0
How/when did you learn about financial assistance available through program (*N* = 283)		
During the interview process	185	65
Program's website	121	43
There was no financial assistance available to me	77	27
On offer day/match day	48	17
Other	33	12
Primary repayment option for federal loans (*N* = 220)		
Standard Repayment Plan	71	32
Graduated Repayment Plan	5	2
Extended Repayment Plan	8	4
Saving on a Valuable Education (SAVE) Plan—formerly REPAYE Plan	72	33
Pay As You Earn Repayment Plan (PAYE)	13	6
Income‐Based Repayment Plan	22	10
Income‐Contingent Repayment Plan	1	0
Loan consolidation	2	1
Do not remember	9	4
Other	17	8
Whether you saw a financial advisor before starting program or taking out loans (*N* = 280)		
Yes	37	13
No	283	87
Helpfulness of financial advising received (*N* = 31)		
Not at all helpful	8	26
A little helpful	10	32
Somewhat helpful	6	19
Very helpful	5	16
Extremely helpful	2	6
Importance of financial advising for future genetic counseling students (*N* = 278)		
Not at all important	0	0
Somewhat unimportant	5	2
Neither important nor unimportant	14	5
Somewhat important	132	48
Extremely important	127	46

Other common funding sources used by participants included personal savings (74%; *n* = 208/280) and family savings (45%; *n* = 127/280). There was not a significant change between the use of personal (*p* = 0.356) or family savings (*p* = 0.586) from 2013 to 2023.

Additionally, just under half of graduates received grants and scholarships (46%; *n* = 128/281). Seventy‐four percent (*n* = 95/127) received their grant or scholarship from their GC program. Of the 41% (*n* = 162/396) of respondents who received a grant or scholarship in 2013, a similar portion received these from their GC program (70%; *n* = 114/162). Additional utilized forms of financial assistance are included in Table 3.

Participants learned about available program financial assistance at different times during the application process. Most (65%; *n* = 185/283) received information during the interview process, 43% found information via the program's website, and 17% did not learn about all available assistance until they were accepted or matched to a program.

In addition to financial assistance, 71% (*n* = 197/279) of participants worked during their GC program, with just under 40% working 6–10 hours per week (Table 3). A similar portion of respondents worked in 2013 (73%; *n* = 293/402; *p* = 0.583) with no significant change in hours worked per week (*p* = 0.844). Of those who held jobs, about half (52%, *n* = 99/192) felt that working during their training program neither helped nor hurt their academic performance. Twenty‐eight percent (*n* = 54/192) stated that working at least somewhat hurt their performance, while 20% said working improved their academic performance. More hours spent working per week was associated with greater negative influence on perceived performance in both 2023 (*r*_s_ = −0.343, *p* < 0.001) and 2013 (*r*_s_ = −0.158, *p* = 0.008), with a stronger negative association in 2023 (0.19 points lower; 95% CI: 0.1 to 0.37 points lower in 2023 than 2013). Overall, 66% (*n* = 124/189) of those who held jobs during their training program said working was somewhat or extremely important in covering their educational expenses in 2023 (Table 3).

Participants also used credit cards to pay for necessary educational expenses (85%, *n* = 239/280), though 64% said that they utilized credit cards without ever carrying a balance (Table 3). Importance of having credit cards available to cover tuition during a GC program was not strongly related to total amount of tuition and fees (*r*_s_ = 0.04, *p* = 0.760).

When considering all sources of education‐related debt, participants graduated with a median total of $50,000 to $75,000, compared with a median of $40,000 to $50,000 in 2013, representing a significant increase from the prior survey before adjusting for inflation (*p* < 0.001), with a 39% increase in median range midpoints, again in the setting of a general 31% increase from inflation (Figure 1b). Total amount of debt from all sources was strongly aligned with total GC program cost (*r*_s_ = 0.52, *p* < 0.001) in 2023, indicating that students who attended more expensive programs took out more loans to finance their education.

### 3.4 Financial advising

Before starting their GC program, most participants did not receive financial counseling (87%, *n* = 243/280; Table 3). Fewer graduates used a financial advisor in 2023 (13%) compared with 2013 (20%; *n* = 79/399), a drop of 7 (95% CI: 0.9–12.1; *p* = 0.025) percentage points. Nearly all (93%; *n* = 259/278) graduates felt financial advising would be somewhat or extremely important for future students, with an almost identical percentage having said the same in 2013 (94%; *n* = 372/396; *p* = 0.686). Of those who did receive financial counseling before starting their program, almost 60% (*n* = 18/31) claimed those services were, at best, only a little helpful, which was again like the sentiment expressed in 2013 (58%; *n* = 40/68; *p* = 0.944). Those who did see a financial advisor before starting their program or before taking out loans tended to view financial counseling for future students as more important (*p* = 0.026). Level of importance of financial advising for future students also did not appear to be related to a respondent's hypothetical borrowing practices if they could start over (*p* = 0.220), and data gave no evidence to suggest that level of hardship caused by student debt differed according to whether a financial advisor had been previously consulted before pursuing a graduate degree (*p* = 0.169).

### 3.5 Impact of graduate student debt

A vast majority of graduates (96%; *n* = 222/232) reported feeling burdened to some degree by their student debt (Table 4). The fraction of those who felt at least somewhat burdened by student debt payments increased 13 (95% CI: 8.3—18.1, *p* < 0.001) percentage points from 2013 (282/342, 83%) to 2023 (222/232, 96%). Of note, total cost of tuition and fees had a strong positive association with degree of hardship caused by student debt for the 2023 cohort (*r*_s_ = 0.28, *p* < 0.001), which was comparable to the association noted in 2013 (*r*_s_ = 0.26, *p* < 0.001). Additionally, degree of hardship caused by student debt in the 2023 cohort was not associated with whether family members provided any financial assistance (*p* = 0.22). In the 2013 cohort, hardship caused by student debt tended to be higher for those who did not have family support compared with those who did (*p* = 0.007). For those from 2023, having familial financial support also did not appear to help relieve the burden one feels from the debt they accrued (*p* = 0.60), though there was some indication (*p* = 0.044) that those in 2013 without family support felt more burdened by debt.

**TABLE 4:** Impacts of graduate student debt (2023).

Question/response	*n*	%
Burden of student debt payments felt by genetic counseling graduates (*N* = 232)		
Not at all burdened	10	4
Somewhat burdened	36	16
Moderately burdened	68	29
Very burdened	72	31
Extremely burdened	46	20
Most influential factor in determining job choice (*N* = 277)		
Location	114	41
Specialty	60	22
Salary	56	20
Co‐workers	14	5
Institution/company	10	4
Personal/professional growth opportunities	12	4
Benefits	6	2
Other	4	1
Paid continuing education opportunities	1	0
Satisfaction of genetic counseling education in regards to investment for professional development provided (*N* = 280)		
Very dissatisfied	5	2
Dissatisfied	5	2
Somewhat dissatisfied	9	3
Neutral	7	3
Somewhat satisfied	42	15
Satisfied	116	41
Very satisfied	96	34
Satisfaction of genetic counseling education in regards to investment for personal development provided (*N* = 279)		
Very dissatisfied	3	1
Dissatisfied	9	3
Somewhat dissatisfied	11	4
Neutral	13	5
Somewhat satisfied	53	19
Satisfied	101	36
Very satisfied	89	32

Regardless of perceived hardship in this study, there was a tendency for those who had no additional help from family members, spouse, etc. to have accumulated a greater total amount of loans needed to fund their GC program (*p* < 0.001). In 2023, an individual without familial financial support had an estimated 62% (95% CI: 55–69%, *p* < 0.001) chance of having to take on more total debt, which was virtually identical to the 63% (95% CI: 57–68%, *p* < 0.001) chance reported in 2013.

**FIGURE 2:**
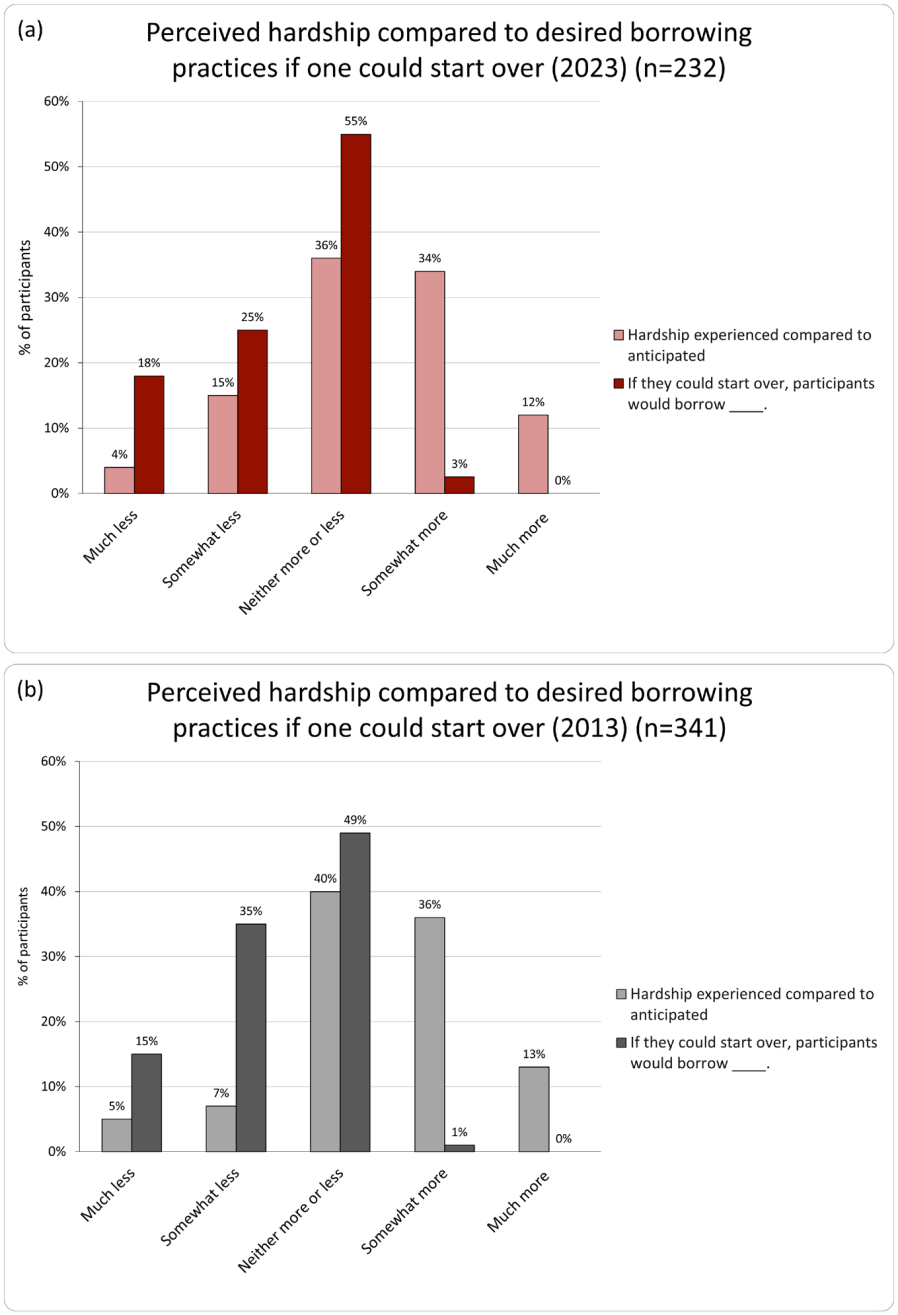
Comparison of perceived hardship (what was anticipated at the time that they took out their student loans) and participant borrowing practices if they could start over in 2023 (a) 2013 (b).

Despite this reported hardship, 60% (*n* = 165/274) shared that they would borrow the same amount if they could start over. However, a greater level of student debt hardship was associated with the desire to borrow lesser amounts in both 2023 and 2013 (*r*_s_ = −0.40 [2013], *r*_s_ = −0.41 [2023]; *p* < 0.001 each year; Figure 2).

A large majority (86%; *n* = 277) also said their student debt did not change their career path post‐graduation, like respondents in 2013 (88%; *n* = 345/392; *p* = 0.354). However, total amount of debt accumulated after graduating from a GC program was significantly higher among those who claimed that their debt altered their career path (*p* < 0.001). The most significant factors influencing job choice for graduates were location (41%, *n* = 114/277), specialty (22%), and salary (20%). Greater amounts of debt aligned with placing more influence on salary (*r*_s_ = 0.17, *p* = 0.005) and to a lesser amount, benefits (*r*_s_ = 0.11, *p* = 0.063).

**FIGURE 3:**
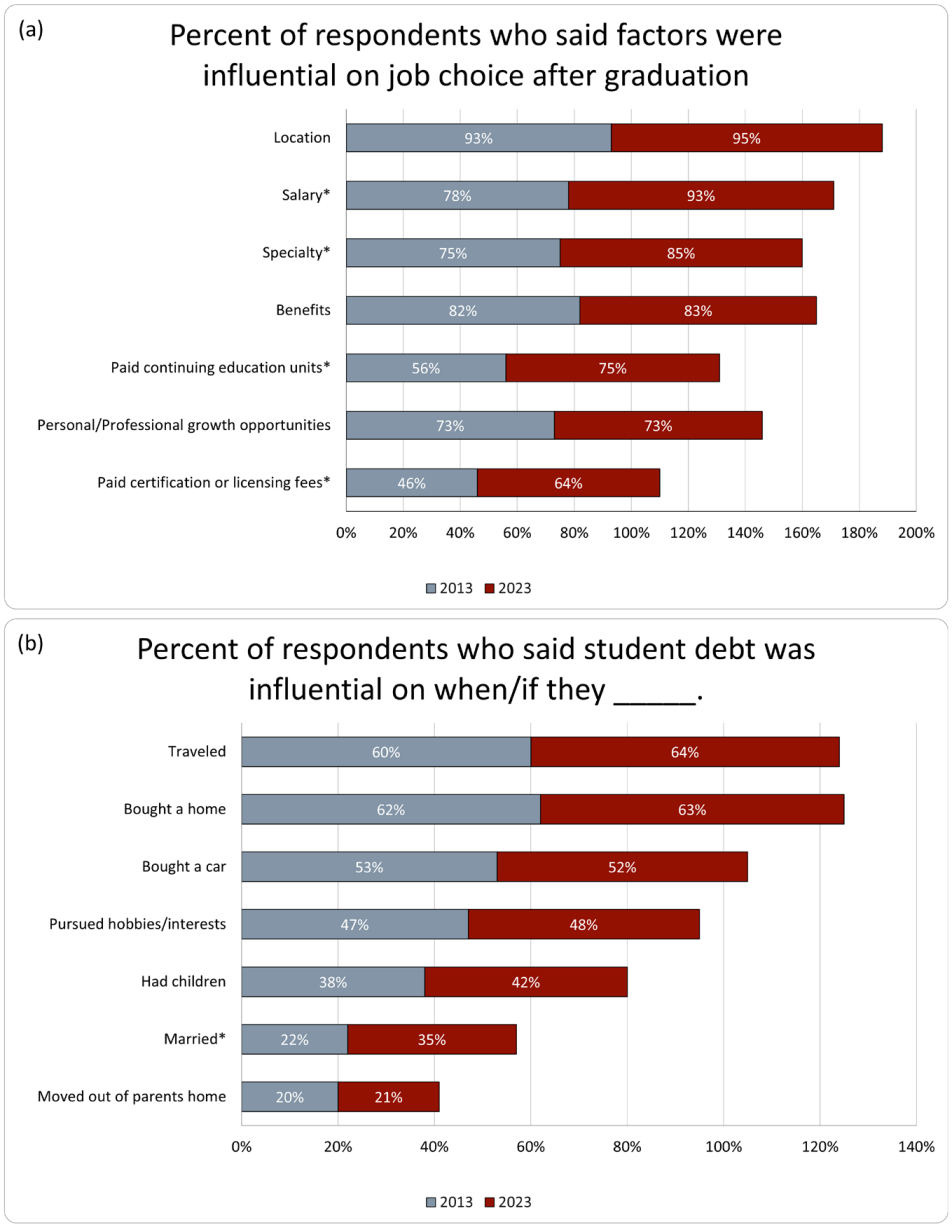
Percent of respondents who indicated that student debt was influential on job choice (a) and life milestones (b) in 2013 (gray) and 2023 (red). *Indicates a statistically significant change from 2013 to 2023 (*p* < 0.05).

Order of job choice priorities remained consistent. Location was rated as most influential by participants in 2013 (58%; *n* = 228/393), followed by specialty (11%) and salary (11%). While there was no significant change in rating location as at least somewhat influential on job choice from 2013 to 2023 (*p* = 0.161), salary increased by 15 (95% CI: 9.7–19.9; *p* < 0.001) percentage points (78% [2013] vs. 93% [2023]), and specialty increased by 10 (95% CI: 3.3–15.5; *p* = 0.003) percentage points (75% [2013] vs. 85% [2023]; Figure 3a).

Most participants (94% [*n* = 253/269]) held full‐time positions, an insignificant change from 2013 (92%; 352/384; *p* = 0.251). Median starting salary for full‐time workers increased ($70,000 to $75,000 [range: <$50,000 to $115,000]), compared with the starting salary in 2013 ($45,000 to $55,000 [range: <$35,000 to $95,000+]) without adjusting for inflation (*p* < 0.001; Figure 1c). This represents an estimated 45% increase between median range midpoints, which is generally in line with the 51% increase reported by NSGC from 2013 to 2023, though direct comparison is made difficult given the nature of the interval responses in this survey. Median current salary also increased before accounting for inflation, to $80,000 to $85,000 (Range: <$50,000 to over $140,000) from $55,000 to $65,000 (Range: <$35,000 to $95,000+) in 2013 (*p* < 0.001).

Of those with student debt, 55% (*n* = 140/254) stated that they were “not at all willing” to take a non‐GC job that paid enough salary to cover their student loans and other living expenses, though more total debt was positively associated with one's willingness to take a non‐GC job if it paid enough to cover these expenses (*r*_s_ = 0.23; 95% CI: 0.11–0.35, *p* < 0.001). Participants did report that their student debt was somewhat or extremely influential on when they bought a home (63%; *n* = 146/233), bought a car (52%; *n* = 124/240), and traveled (64%; *n* = 154/241). The percentage of respondents who said student debt was influential on various lifestyle factors remained mostly consistent, apart from when/if they got married being more influenced in 2023 (22%; *n* = 78/362 in 2013 to 35%; *n* = 82/234 in 2023; Figure 3b).

Level of total debt and degree of hardship caused by student debt compared with what was anticipated did not strongly differ according to marital status (*p* = 0.530 and *p* = 0.089, respectively). Total number of dependents also did not appear to be associated with the level of hardship caused by student debt (*r*_s_ = −0.033, *p* = 0.617).

Although certain GC program graduates' post‐graduation choices were influenced by their student debt, approximately 89% of respondents stated they still would have attended the same GC program given enough financial resources (*n* = 205/231). A similar portion of participants (90%) reported the same in 2013 (*p* = 0.665). Most respondents were “satisfied” or “very satisfied” with their GC program regarding professional development (76%) and personal development (68%; Table 4). Level of satisfaction with professional development and the degree of student debt hardship were not strongly related (*r*_s_ = −0.11, *p* = 0.088), though satisfaction with personal development was lower when student debt hardship was high (*r*_s_ = −0.14, *p* = 0.030). Eighty‐four percent (*n* = 192/230) of respondents felt that their GC education was at least equally as beneficial compared with what they expected given the loans they took out for it.

## 4 DISCUSSION

Despite the increase in salaries over the last decade, GC program graduates are still reporting high financial burden and more hardship than expected from their student loans. Many patterns appear to be strikingly similar across 2013 and 2023 cohorts in terms of program, professional, and life choices. These findings regarding impacts of student debt have been highlighted among other allied health professions, with the PT population specifically reporting high levels of burden/anxiety, limitations on job choice and program selection, and decisions to delay or forgo certain life milestones due to their student debt (American Physical Therapy Association, 2022).

While there were some small shifts in participant demographics, this cohort still represented a largely homogenous group (87% female, 81% White). This is relatively reflective of demographic information reported in the 2023 NSGC PSS (93% female, 86% White). Given the modest increase in diversity but overall persistence of homogeneity within this study and the overall profession, there may be greater ongoing barriers to entering the field faced by individuals from underrepresented and/or low SES groups. Additional resources to address student debt could increase financial access, though additional research is needed to further assess barriers.

Financial help from family members did not appear to reduce degree of hardship reported by respondents in 2023 as it did in 2013. It is unclear whether parents and family members in this cohort could not provide as much financial help, or whether the overall rise in educational costs has outpaced the degree of financial help that families can provide. However, a 2023 analysis did find that most college students had unmet need and experienced gaps between what their families could pay and their college costs (Institute for Higher Education Policy, 2023).

Many respondents anecdotally shared feelings of regret regarding cost of the programs they chose to pursue/rank. Several also said that financial advising likely would have had little impact on their choices for many reasons, including limited available financial information prior to applying/matching and feeling pressured to prioritize an acceptance/match over cost. These sentiments were consistent with survey findings indicating that financial advising did not impact hardship caused by student debt, despite graduates noting importance for future students.

Multiple graduates reflected on their experiences, sharing:

I didn*'*t feel that I could properly assess how much debt I would need to take on for any of the programs I applied to. It was very hard to find any concrete information about student loan options/scholarships. None of the programs provided transparent information and I did not know how much “student aid” I would get until after I was accepted.

No amount of financial counseling would have let me make a different choice in regard to my grad program though, because the match system does not allow you to really have a choice*…*. Not to mention that the programs are so selective that it doesn't feel like much of a choice but to go.

A possible explanation for these findings may be more specifically related to when students receive information regarding program costs and aid, the Genetic Counseling Admissions Match, and if/when a financial advisor is consulted. The Accreditation Council for Genetic Counseling (ACGC) required an “estimation of all costs (tuition, fees, etc.) related to the program” to be available online by August 1, 2024 (GC Education, 2023, p. 14); however, this stipulation did not specifically include financial assistance options/availability. Most respondents in this study learned about available financial assistance during the interview process, and another portion did not learn about some or all information until they were accepted or “matched” to a program and in binding commitment to attend (https://natmatch.com/gcadmissions/overview.html). Lack of full financial transparency throughout the entire admissions process limits applicant consideration of financial factors and could impact financial wellness after graduation.

A vast majority once again reported feeling burdened by their student debt payments, though this may not be surprising given the current financial climate, reported debt loads, and starting salaries among this cohort. Expert recommendations have traditionally said students should not borrow more than 8%–10% of their projected gross monthly income or 20% of their expected discretionary income. The Institute of Student Loan Advisors leadership has suggested capping the limit even lower, at 10% of discretionary income (Carrns, 2024). Most recently in 2022, the Consumer Financial Protection Bureau (CFPB) generally recommended that students not borrow more than their expected starting salary one year after graduation with monthly payments limited to 10% of gross monthly income (Consumer Financial Protection Bureau, 2022).

Personal finance websites like SmartAsset.com can be used to estimate monthly payments and feasibility of repayment based on total debt, salary, loan interest rate, and repayment plan (https://smartasset.com/student‐loans/student‐loan‐calculator#JJX3d6vZFm). This calculator cannot use median ranges, so a mean for the present sample was calculated using the weighted average of the amounts reported by participants. This is an approximate weighted average for total debt, as the value for the last category (e.g., $120,000 or more) was an interval (e.g., $10,000) higher than the listed value (e.g., $130,000). When applying the 7.05% interest rate (as of July 1, 2023) for Graduate or Professional Direct Unsubsidized Loans to the federal limit of $41,000 across a 2‐year program, the 8.05% rate for Federal Graduate PLUS loans to the remaining $34,325 of the average student debt from this data, a starting salary of $75,325, and the reported approximate weighted average for student debt of $64,366 with a standard fixed‐rate 10‐year repayment plan, the calculator indicates a monthly payment of $894, which equates to about 14% of the average gross monthly income reported in this study. While the total student debt load reported by respondents does not exceed the average starting salary, estimated monthly payments at these interest rates exceed the more liberal recommendation that payments do not exceed 10% of gross monthly income. These calculations support the hypothesis that recent GC program graduates are experiencing similar hardships to their counterparts from 10 years ago. These results indicate ongoing high debt loads and burden from student debt, potentially resulting in the reported impacts on professional and personal life choices.

### 4.1 Practice recommendations

Previous efforts within genetic counseling and other medical professions to raise funds for students have included philanthropic initiatives to increase financial access, with many programs and scholarships being funded by GC program alumni. Given the persistent financial hardship caused by student debt for graduates over the last decade, additional research is needed to determine whether this model is sustainable as a funding source for future students. Outside of alumni donations, programs may consider other initiatives to help decrease associated costs for students, such as allowing flexibility within the program structure/curriculum for jobs, an important financial resource for most of our participants. Additionally, programs may put effort toward increasing awareness of grants and other financial resources, including financial counseling regarding program costs, budgeting, loan repayment, and/or forgiveness options. Increased financial transparency from programs early in the application process is also a way to support informed financial decision‐making for prospective applicants.

### 4.2 Future directions

While this study provides insights into how student debt impacts professional and personal choices for a cohort of recently graduated GCs, this may not be generalizable to the entire field, especially for programs outside of North America. Future studies may also evaluate the impact of student debt on other age groups, whether student debt similarly or disproportionally impacts underrepresented groups within the profession, and how SES status affects how this debt impact is perceived. Additionally, future studies may further explore the meaning of “burden” and its interpretation by respondents, as well as negative financial impacts as they pertain to graduate students in different economic landscapes and time periods and those with differing backgrounds (including SES and demographics).

Future studies may also evaluate timing and utility of financial advising for incoming GC students, availability of financial information from programs, and financial implications of the Match program contract.

While ongoing discussion about legislative policies to reduce the financial burden of higher education at the national level continued during the time of this survey, differences in political perspectives and changes in elected leaders create uncertainty regarding how effective and sustainable federal programs may be in the future. Available federal loan repayment options and forgiveness programs are subject to changing legislation, which is reflected in options utilized by this study cohort. Future studies may evaluate how the availability of federal programs impact the financial wellness of graduates in the years to come.

### 4.3 Study limitations

The survey was limited to GC program graduates of the last five years to help limit confounding economic variables, as individuals in this group started their program and/or entered repayment during the COVID‐19 pandemic and associated economic and repayment policy changes. However, given that this study evaluated financial factors, including costs, income, debt load, and related trends over time, there are inherent limitations in interpretation due to the inability to control for all larger socioeconomic forces, such as pandemics, industry contractions, and impact of healthcare system cost‐reductions, as well as smaller industry‐specific forces like fluctuations in non‐clinical GC roles. Additionally, cost, debt, and income comparisons in this study were not directly adjusted for inflation, thus limiting cost comparisons between time periods. Future studies may further evaluate direct financial comparisons between more limited timeframes with inflation adjustment to make stronger conclusions regarding cost, debt, and income changes over time.

Additionally, the survey was sent to individuals who were members of NSGC, whose training program directors had updated contact information for recent graduates, and users on social media who accessed the survey and fell within participant demographic requirements. This approach may limit generalizability by excluding individuals who graduated from 2013 to 2017 and those who have left the field. It is also possible that individuals who opted to take the survey were more motivated based on personal financial circumstances, though this cannot be determined from the data. Additionally, the five‐year timeframe may limit generalizability, particularly to those who entered the field later in life and may have experienced different life milestones compared with younger graduates. Participants were asked to recall retrospective information, which may also result in errors or inconsistencies in reporting. This study also evaluated how student debt has impacted graduates of GC programs, and it cannot be used to draw conclusions regarding cohorts who chose or were not able to pursue the field due to financial barriers.

## 5 CONCLUSIONS

Despite higher salaries, these results support that most financial challenges experienced by recent GC graduates have not changed significantly from a decade ago. These findings are also generally reflective of rising costs within higher education and ongoing economic challenges. Many who have successfully pursued this profession in recent years also had financial help from family members and/or little to no undergraduate student debt, yet a majority still reported feeling burdened by student debt and more hardship than initially anticipated. Given that student debt‐to‐income ratios for GCs continue to outpace recommendations from financial experts, we propose ongoing discussion and action to address the financial burden on GCs following graduation and financial barriers faced by prospective students, especially as we look to increase access and diversity within the profession.

## AUTHOR CONTRIBUTIONS

HG led survey adaptation and development, data analysis, and overall writing and editing of this manuscript*. All authors (HG, EP, ML, and AK) contributed substantially to the design of the study, drafting, and revising of the manuscript.

## CONFLICT OF INTEREST STATEMENT

Holly Greer, MGCS, CGC; Elizabeth Petty, MD; Michael Lasarev, MS; and Ashley Kuhl, MS, CGC, do not have any financial or non‐financial conflicts of interest to disclose.

## ETHICS STATEMENT

Human studies and informed consent: Informed consent statements were listed on the first page of the survey. Participant anonymity was preserved and identifying information has been excluded from this manuscript.

Animal studies: No non‐human animal studies were carried out by the authors for this article.

## Data Availability

The data that support the findings of this study are available from the corresponding author upon reasonable request.
